# Microfluidic production of silver nanoparticles demonstrates ability for on demand synthesis of a wide size distribution of particles

**DOI:** 10.1007/s11051-024-05944-1

**Published:** 2024-02-21

**Authors:** Katelyn J. Langguth, Sara Maccagnano‑Zachera, Joshua Heinemann

**Affiliations:** Montana Microfabrication Facility, Montana State University, Bozeman, MT 59717, USA; Montana Nanotechnology Center, Montana State, University, Bozeman, MT 59717, USA; Montana Nanotechnology Center, Montana State, University, Bozeman, MT 59717, USA; Image and Chemical Analysis Laboratory, Montana State, University, Bozeman, MT 59717, USA; Montana Microfabrication Facility, Montana State University, Bozeman, MT 59717, USA; Montana Nanotechnology Center, Montana State, University, Bozeman, MT 59717, USA

**Keywords:** Nanoparticle, Microfluidics, Chemical synthesis, Silver, Tunable size control

## Abstract

Silver nanoparticles (AgNP) can help prevent infection of virus and bacteria. The size and morphology of AgNP can be crucial to function, with smaller nanoparticles (< 20 nm) able to penetrate the cell wall. This is significant as oxidative stress and genotoxicity are associated with some sizes and coatings of AgNP, contraindicating the use of AgNP to reduce infection. We present evidence that a microfluidic chip can synthesize larger sizes and distributions of AgNP from the nano-to-micro size range. We show results from a microfluidic mixing chip that can produce a wide range of nano-to-micro size (~ 24–400 nm) AgNP. Synthesis is based on a modified Turkevich method, using a single-step AgNP synthesis on the microfluidic chip using two chemical components, trisodium citrate (NaCit) and AgNO_3_. To make AgNP more accessible, we describe the microfluidic chip and conditions capable of synthesis. We also describe how modification of flow rate and chemical reagent concentration change particle diameter during production. In our experiments, we found that AgNP production created a visible adsorption line in the microfluidic device, possibly owing to AgNP surface interaction at the polydimethylsiloxane (PDMS) interface. We characterize these particles with dynamic light scattering (DLS), transmission electron microscopy (TEM), and scanning electron microscopy (SEM). Based on optical light microscopy, we hypothesize that AgNP formation primarily occurs at the interface between the two chemical reagent streams. We also conclude that AgNP size increases could be due to interaction with the PDMS surface, which is known to be porous. Future work will help to understand how surface interaction may influence the formation of larger particles.

## Introduction

Nanotechnology innovations have surged in the past twenty-four years due to the accelerated development of the field from the National Nanotechnology Initiative [1]. The basis of this technology involves the use of atoms or particles sized between 1 and 100 nm [2]. This research is notable because nanoparticles are found to exhibit properties that differ from their bulk counterparts and provide a fundamental material from which to build upon [2, 3]. The commercial uses for nanoparticles are greatly increasing to include catalysts, microelectronics, drug carriers, and more [4]. AgNP has been used in a wide range of fields from orthopedics [5], dentistry [4, 6], textiles and plastics [6], photovoltaic technology [3], and information processing devices [7].

Silver is known to be a non-toxic metal with broad antibacterial properties like copper [8, 9]. This property gives AgNP the potential to overcome broad-spectrum microbial resistance making them potentially promising antibacterial agents [10]. The broad spectrum bactericidal or bacteriostatic activity has been found beneficial for the prevention of the emergence of antibiotic-resistant bacteria when placed within the body [9]; however, some questions of the safety of smaller (< 20 nm) AgNP persist [11, 12]. The slow-release antiseptic activity of AgNP is thought to be the primary driver for the production and incorporation of AgNP into pharmaceuticals [9–11]. The enhanced antibacterial properties of nanoscale silver (compared to bulk) make it a valuable product in water disinfection, fungal infections, biofilm prevention, or incorporation into clothing and linens [3–6, 13, 14]. However, when synthesizing particles, consistency and reproducibility are important because the size and morphology of the particles affect their properties [12, 15–17]. Therefore, the replicable production of high-quality AgNP is of the utmost importance.

The synthesis of AgNP can be achieved through various methods, but microfluidic chips offer a simple, efficient, and scalable alternative [17, 18]. Microfluidic devices or chips named from their fabrication from microchip technology can be digitally controlled and have a variety of useful functions applicable to chemical nanoparticle synthesis. Several groups have conducted predictive simulations of the critical parameters of nanoparticle synthesis on microfluidic devices [19, 20]. Incorporating multiple microfluidic functions together could allow for chemical synthesis and particle size separation using inertial forces [21–23]. This work suggests microfluidic nanoparticle synthesis provides a tunable environment capable of sequential processing or parallel synthesis. A microfluidic device uses minimal amounts of sample and generates minimal waste, creating a low-cost, reproducible, and precise method for synthesizing AgNP [24–26]. The AgNP demonstrated in this work are synthesized using a modified Turkevich method via a NaCit reduction [27]. In Turkevich synthesis, cationic metal (e.g., Au or Ag) is both reduced, and nanoparticle growth is capped using trisodium citrate (NaCit) [28]. The method is desired and known for its use of nontoxic substances and minimal waste critical for applicability for in vivo study. AgNP has been demonstrated to kill 650 different disease-causing organisms that are found in the body [6]. AgNP has a high stability and citrate is a non-toxic capping agent for the particles, creating a safe and controlled synthesis using microfluidics [27–30]. The advantages of using a microfluidic device include efficient mass and heat transfer, efficient mixing [16], and minimal waste.

In this study, a microfluidic device was used to synthesize AgNP through the reduction of silver nitrate (AgNO_3_) using NaCit in a minimalist one-step method. The synthesis was tested under various flow rates and temperatures to determine the best conditions for AgNP production. The results suggest chip material, flowrate, and chemical concentrations can be modified to access a wide range of particle sizes on the same device.

## Materials and methods

### Materials

SU-8 3050 photoresist was purchased from Kayaku Advanced Materials (Westborough, MA, USA). The photomask was obtained from CAD/ART Services (Bandon, OR, USA). The Silicone Elastomer Base and Silicone Elastomer Curing Agent (SYLGARD 184) were purchased from Amazon (Seattle, WA, USA). Tri-Sodium citrate dihydrate (NaCit), Hi-AR/ACS (Na_3_C_6_H_7_•2H_2_O, Mw = 294.10 g/mol) and silver nitrate, ACS ( AgNO_3_, Mw = 169.87 g/mol) were obtained from HiMedia Laboratories (Kennet Square, PA, USA) and Chemsavers (Bluefield, VA, USA), respectively. Sodium dodecyl Sulfate was purchased from Millipore Sigma (Burlington, MA USA).

### Fabrication of microfluidic device

The mold for the microfluidic device was created using SU-8 3050 photoresist by following the processing guide from Kayaku Advanced Materials. The photoresist was spun onto a plasma-cleaned glass microscope slide with a final thickness of 60 μm. The slide was exposed using an ABM contact aligner lamp with PL-360-LP filter and photomask designed in AutoCAD 2021 (Autodesk, San Francisco, CA). The completed mold was then coated in SDS-PAGE surfactant and polydimethylsiloxane (PDMS) in a 10:1 mixture of Silicone Elastomer Base and Curing Agent was poured onto it. The mixture was degassed and cured overnight at 65 °C. The PDMS was then removed from the mold, and the edges were cleaned using a razor blade. The port holes were punched out using a hollow metal syringe tip. The device was then assembled by O_2_ plasma cleaning a clean glass slide and the PDMS before pressing them together. The completed device was heated at 65 °C for 5 min on a hot plate. The microfluidic device was analyzed by optical profilometry on a Profilm3D by Filmetrics using white light interferometry. The scan was made with a 5 × objective in white light mode with 3 × scan averaging.

### Production of AgNP

For positive control experiments, batch synthesis of silver nanoparticles using a modified Turkevich method was conducted to first make control particles. These particles were imaged via field-emission scanning electron microscopy. Briefly, particles were synthesized using 1 mM solution of AgNO_3_ by bringing 35 mL to a boil in a 100 mL beaker and adding 5 mL of 100 mM NaCit and boiling for 10 min. A characteristic change in the color of the solution appeared as the colloidal silver nanoparticles formed, wherein the solution turned from clear to a yellowish color. Samples of 1 μL were then removed by pipette and pipetted up and down on a piece of silicon wafer coated with hexamethyldisilazane (HMDS). The diced wafer was then loaded into the SEM for imaging.

**Fig. 1:**
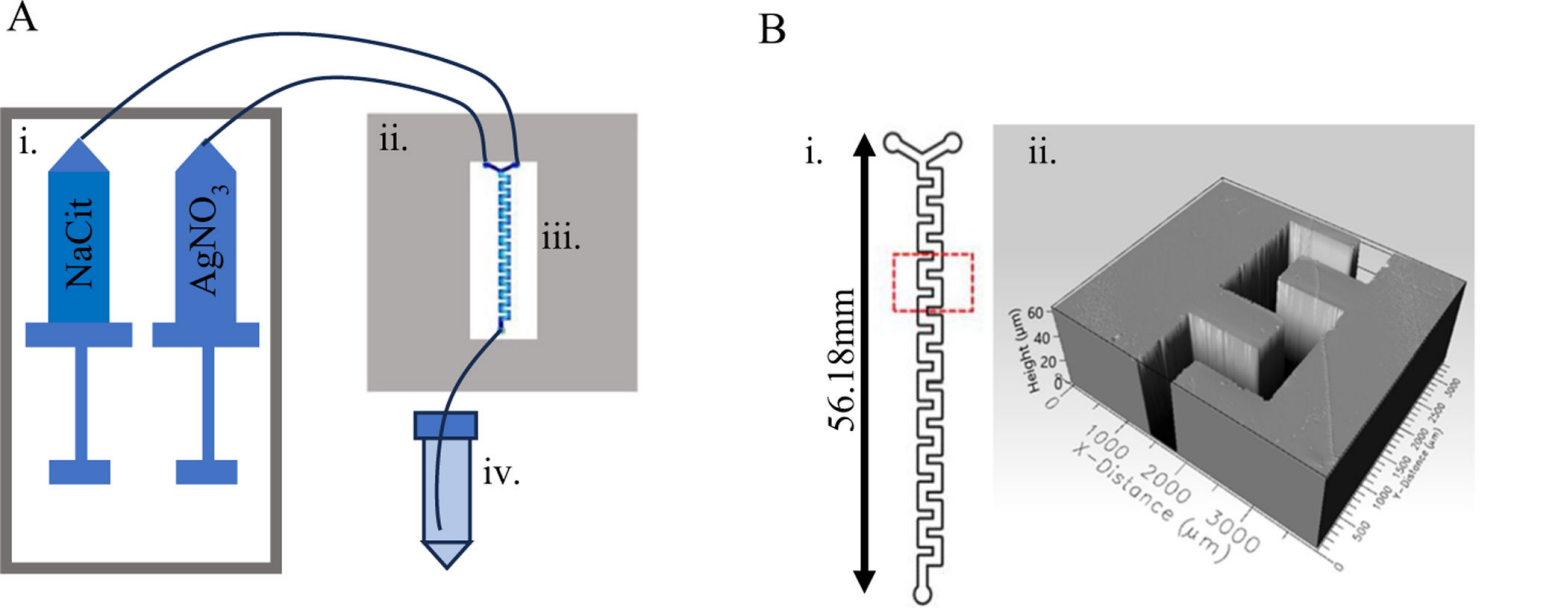
Microfluidic system setup and theory of operation. **A** Setup of microfluidic operation fully assembled, i. dual syringe pump where one syringe contains NaCit and other syringe contains AgNO_3_, ii. hotplate, iii. microfluidic chip, and iv. Centrifuge tube for collection; **B** design of microfluidic chip. The fluidic channel width is 500 μm width, 60.01 μm height, with a 56.18 mm total length from inlet to outlet ports, red box shows the approximate location of ii. an optical profilometer image of the microfluidic channel in PDMS

The microfluidic production of AgNP utilized a 2 mM AgNO_3_ solution and a 37.5–75 mM NaCit solution. Reaction solutions were prepared in 3 mL syringes. The syringes were connected to the input and outlet ports of the microfluidic device with PEEK tubing 1/32″ OD and 0.08″ ID. The syringes are driven using a dual syringe pump. The pump was run at a flow rate between 0.5 to 3 μL/min while the chip was placed on a hot plate at 85 °C (Fig. 1).

To test continuous microfluidic production of AgNP, the dual syringe pump was run between a flow rate of 0.5 and 3 μL/min at a fixed chemistry of 37.5 mM NaCit (syringe 1) and 2 mM AgNO_3_ (syringe 2). The sample from the outlet channel was collected in a microcentrifuge tube (Fig. 1A) until there was at least 1 mL of sample. After each experiment, the device was flushed with 3 mL of DI water to prevent crystallization buildup in the channel. The production process was repeated until a silver film could be seen in the channel, after which the channel was imaged and cleaned with a 10%(v/v) nitric acid solution and deionized water (DI) at 50 °C. This would remove all visual evidence of silver and return the chip to its original condition. The operation of the chip was repeated for several weeks to determine the longevity of the device. Devices tended to delaminate at the (PDMS–glass) interface after 10–20 mL of reactants.

### Dynamic light scattering

Dynamic light scattering (DLS) was done by UV–Vis spectrophotometer. Absorbance curves for samples of AgNP were built using a 300–1000 nm wavelength sweep. The peak absorbance at 420 nm indicates the presence of AgNP.

### Scanning electron microscopy and transmission electron microscopy of AgNP

Scanning electron microscopy (SEM) was used to observe the success and shape of the AgNP production (SEM; Supra VP 55, Zeiss, Germany) using a 1 kV beam voltage and 5 mm working distance. A micropipette was used to draw 1μL of sample from the centrifuge tube. The collected AgNP samples were first placed as a small drop onto a cleaved silicon wafer coated with HMDS and dried into a spot of ~ 1 mm. The high-resolution images provide detailed information on the morphology, size, and distribution of the AgNP produced, as well as to assess the quality of the production process. Transmission electron microscopy (TEM) was used to observe the success and shape of the AgNP production (TEM; LEO 912, Zeiss) using 120 kV, Proscan 2048 HSC. Information on the morphology, size, and distribution of the AgNP was produced.

### AgNP image analysis

Images were analyzed using imageJ 1.53t software from the National Institute of Health. Briefly, images were opened in imageJ and the scale for the image was set by using the “set scale” in the Analyze tab. A straight line was drawn over the scale bar and the number of pixels to nanometer length was set. Once the ratio (pixel/nm) was set in the image, measurement of the individual particle circumference was possible. Results were exported to CSV and imported into Excel for generation of box and whisker plots. The box and whisker plots use boxes to show how the distribution of particle size is distributed into quartiles. The boxes have lines that extend vertically called whiskers whose lines indicate variability outside the upper and lower quartiles.

## Results and discussion

### Microfluidic device characterization

The microfluidic device was made of PDMS, and after releasing from the SU-8 mold, profilometry images were taken of the channels. Figure 1A shows the microfluidic setup, which consists of a dual syringe pump, two syringes one with NaCit and one with AgNO_3_. Peek tubing connects the syringes to the microfluidic device (iii.) resting on a hotplate (ii.) with a collection tube (iv.) to collect the AgNP as they come off the chip. Figure 1B is a profilometer image of the PDMS channel. The channel was found to be 500 μm in width and has a step height of 60.01 μm by white light optical profilometry. The total flow path length was 56.18 mm with inlets and outlets 2 mm in diameter, and holes were punched with 1 mm hole punch through the PDMS.

### Production of AgNP

**Fig. 2:**
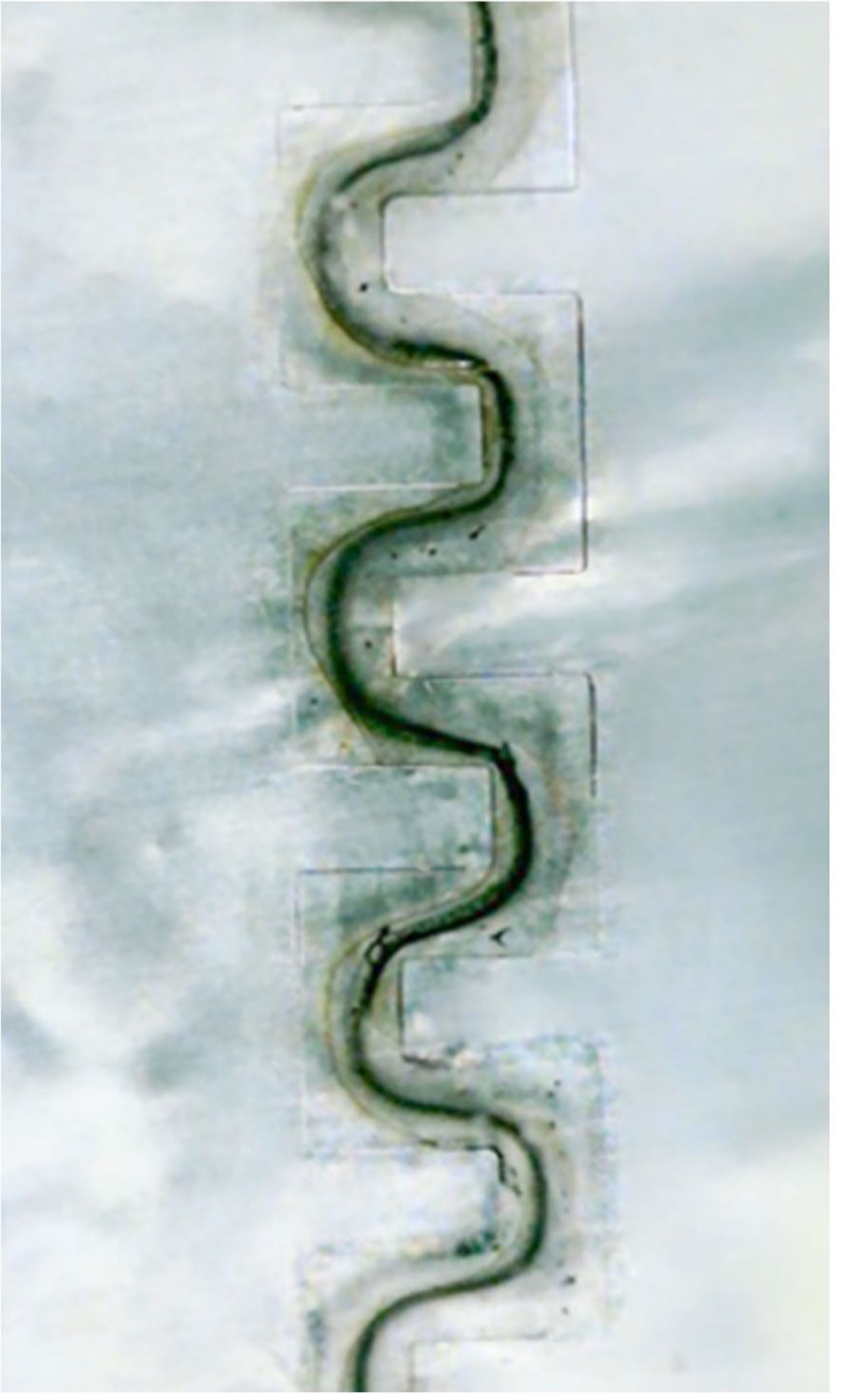
Image of the microfluidic device from optical microscope after 5 h of operation at 0.5 μL/min flowrate with 2 mM AgNO_3_ and 37.5 mM NaCit

**Fig. 3:**
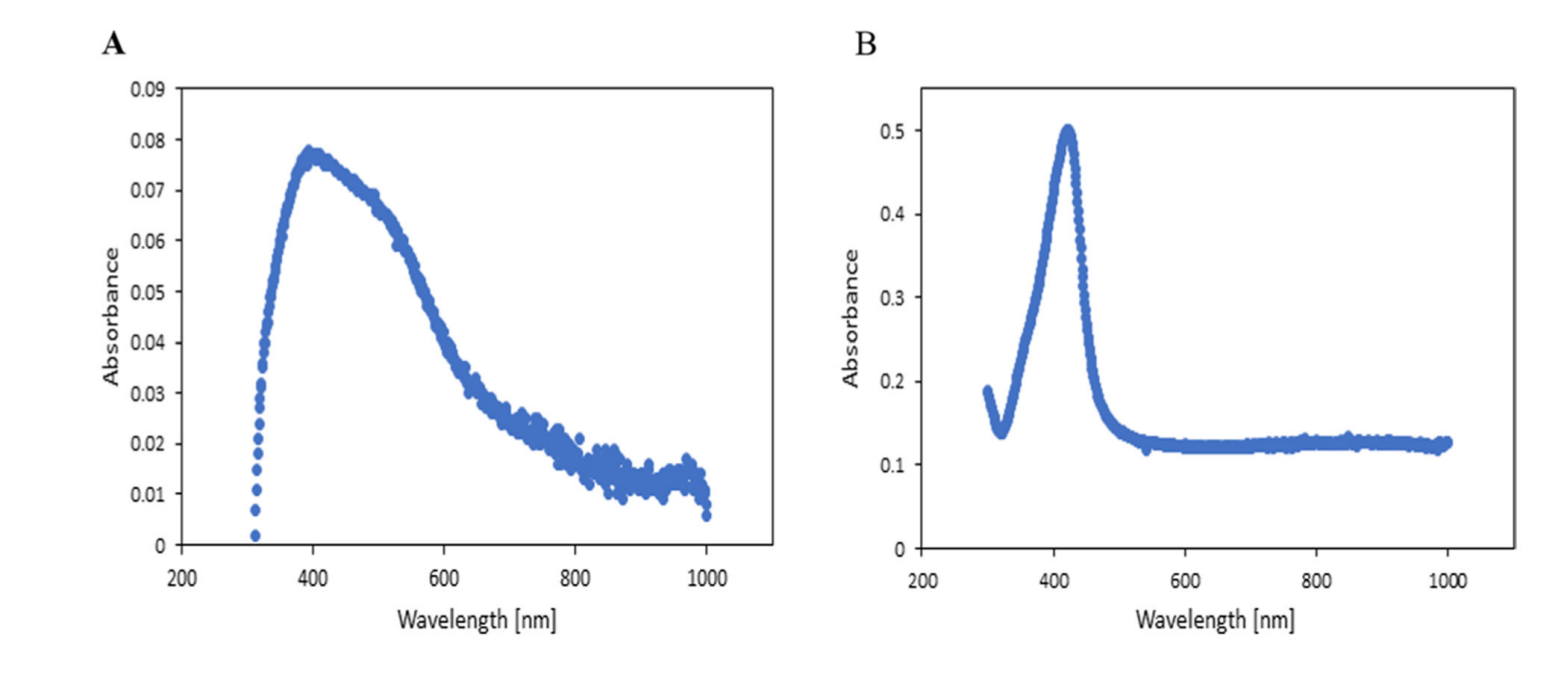
Microfluidic AgNP using UV–Vis spectrophotometer DLS absorbance curves for two samples of AgNP run through a 300–1000 nm wavelength sweep. **A** 1 μL/min flowrate at inlet concentrations of 37.5 mM NaCit and 2 mM AgNO_3_ with a peak absorbance 0.08 AU; **B** 0.5 μL/min flowrate at inlet concentrations of 37.5 mM NaCit and 2 mM AgNO_3_. with a peak absorbance of ~ 0.51 AU. The peak absorbance at 420 nm indicates the presence of AgNP

**Fig. 4:**
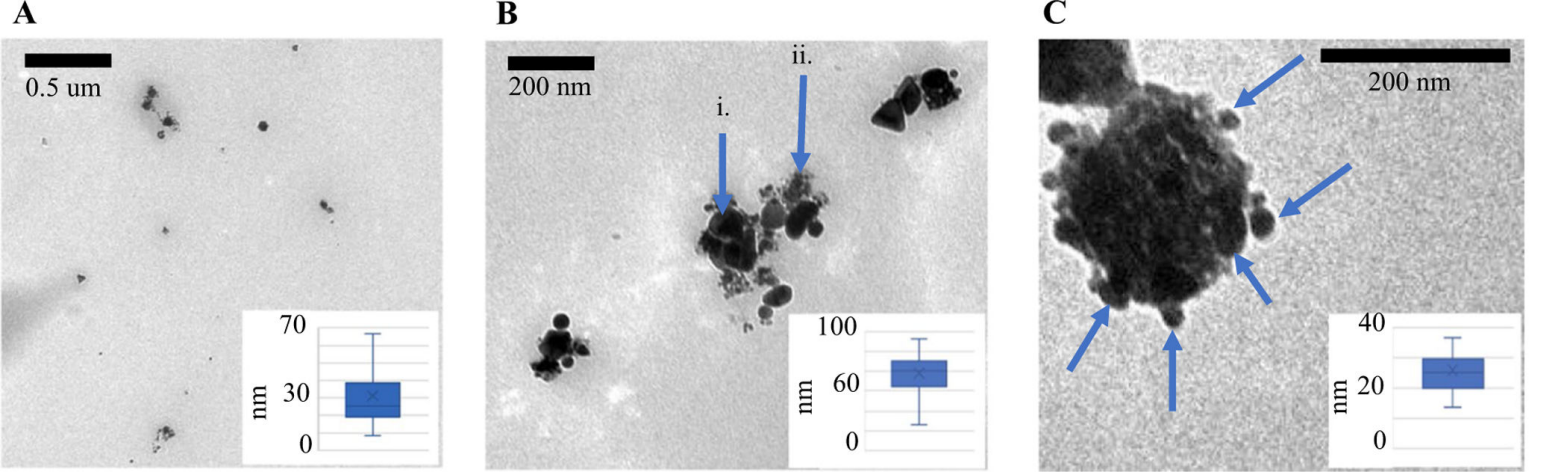
TEM images of the AgNP with box and whisker plots of particle diameter (nm). **A** TEM image from the batch control with 37.5 mM NaCit and 2 mM AgNO_3_, box **B** microfluidic AgNP with the same final concentrations of 37.5 mM NaCit and 2 mM AgNO_3_, two size distributions of AgNP, (i.) larger AgNP plotted in box and whisker plot, (ii.) cluster of smaller AgNP, **C** AgNP coated with smaller AgNP (arrows), box and whisker plot represents the size of smaller AgNP

**Fig. 5:**
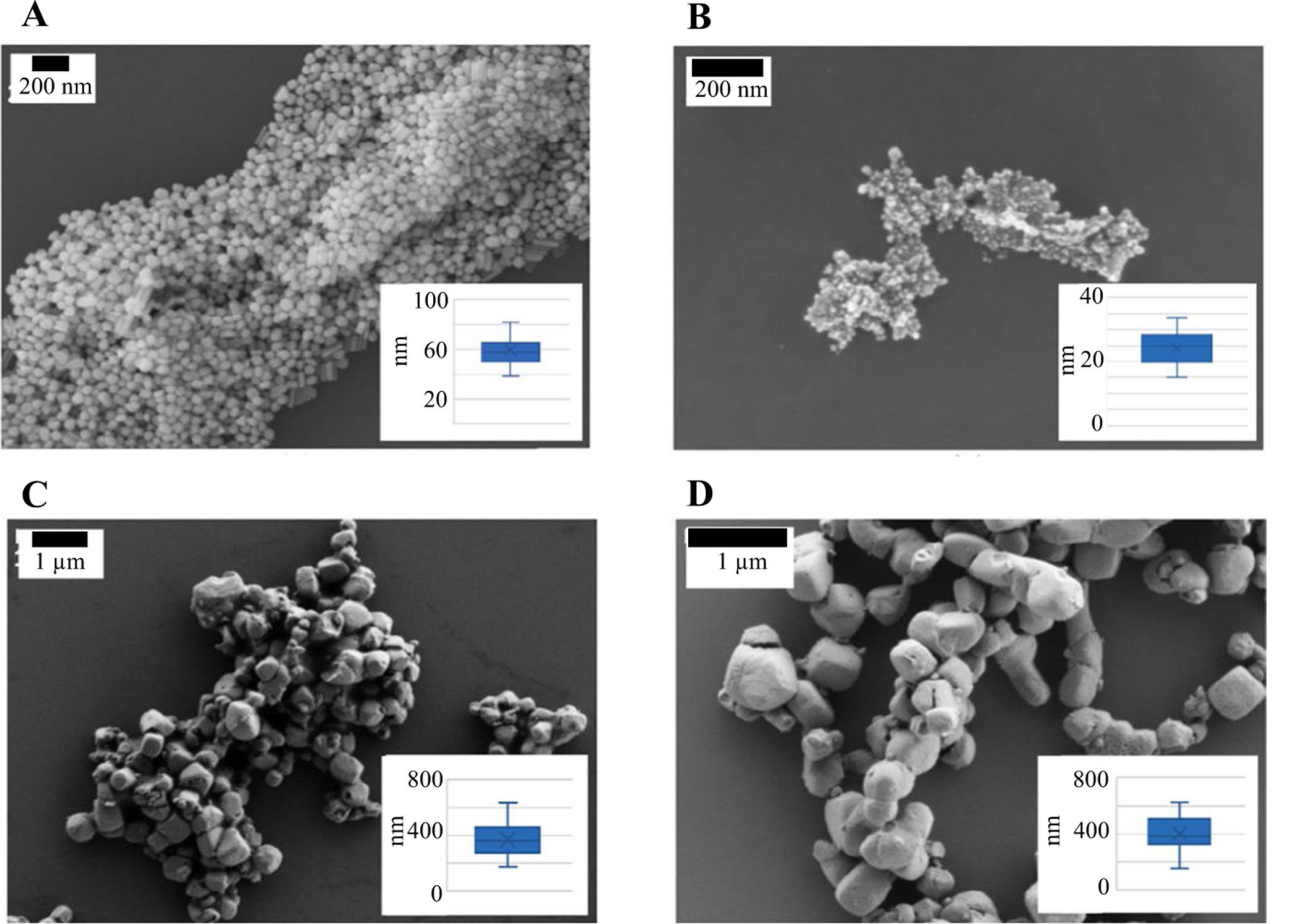
SEM images of the AgNP, box, and whisker plots represent AgNP diameter in respective sample. **A** Positive control optimized batch experiment 37.5 mM NaCit and 1 mM AgNO_3_ and **B** microfluidic AgNP using 37.5 mM NaCit and 2 mM AgNO_3_, at 3 μL/min flowrate. **C** The microfluidic device method with 75 mM NaCit and 2 mM AgNO_3_ at 1 μL/min flowrate. 75 mM NaCit and 2 mM AgNO_3_. **D** A repeated experiment with the same condition as C

A syringe of 2 mM AgNO_3_ solution and a syringe of NaCit (37.5 or 75 mM) solution was prepared. The syringes were connected to the input ports of the microfluidic device and the syringes were placed onto a dual syringe pump. The pump was run at a flow rate of 1 μL/min or 3 μL/min while the device was placed on a hot plate at 85 °C. Higher temperatures would lead to solutions boiling as they traversed the microfluidic chip, leading to bubbles forming in the channel. Evidence of AgNP production was observed using optical microscopy (Fig. 2.), DLS (Fig. 3), TEM (Fig. 4), and SEM (Fig. 5). The production of AgNP was checked after a collection period of 1 mL of solution. It was observed that at flow rates of > 1 μL/min (total flowrate) the presence of particles would not be detectable by DLS. We observed the NaCit/AgNO_3_ solution needed sufficient time at high temperature to achieve high enough AgNP formation to be detectable by DLS. In this context, the time was dictated by flowrate; therefore, the solutions that experience less time on the heated microfluidic chip at 3 μL/min had lower overall AgNP concentrations compared to 1 μL/min. This is shown in Fig. 3A and B, where A shows the AgNP formation at 3 μL/min with a lower absorbance peak (0.08AU) compared to Fig. 4B using the same solutions at 1 μL/min flowrate (0.51AU). When DLS was insufficient for the detection of particles, SEM could still be used successfully when there were < 1000 NP per 1 μL solution. This was accomplished by drying a droplet of solution on an HMDS-coated silicon wafer. A 1 μL droplet was pipetted on the wafer and it was dried into a very small diameter “circular spot” (< 1 mm) with the highest concentration of AgNP in the middle. DLS was sufficient for the detection of AgNP at flow rates between 0.5 μL/min and 1 μL/min (total flow rate) but was often undetectable at 3 μL/min. The detection of AgNP in Fig. 3A was only possible when the chemicals were < 1 month old as the chemicals slowly lost their ability to create AgNP over time, possibly due to chemical oxidation. Optical inspection revealed a thin line of silver (silver band in luster) seen by the naked eye, clearly visible after ~ 5 mL of reactants had been run through the chip. The silver band could not be washed away with deionized water or with flushing the channel with up to 10 × flow rates. The line remained unperturbed unless it was treated with 10% nitric acid at 50 °C. As is shown in Fig. 2, a distinct thin line of silver was visible running through the middle of the channel.

### Silver nanoparticle characterization

The AgNP was first characterized by DLS using a Biomate 3S UV–Visible Spectrophotometer from Thermo Scientific. The spectrophotometer was used to scan a 300–1000 nm wavelength sweep to plot the observed absorbance values of the samples. Figure 3 shows the results from two samples of AgNP collected from the device with the same chemical feed ratios, but different flow rates yield different concentrations of nanoparticles. Independent of the concentration, both plots show a maximum absorbance from 400 to 420 nm indicating the presence of AgNP. These values are consistent with literature, where absorbance values of AgNP created from silver nitrate generally have a peak absorbance value around 400 nm and expand to about 435 nm for larger particles [30]. It was determined that a higher flow rate correlates with a lower production of NPs and that a lower flow rate correlates with a higher production of NPs as seen in Fig. 3. This indicates that the relationship between flow rate and AgNP production is inversely proportional. Figure 3B represents the maximum absorbance value of AgNP production on the microfluidic chip (0.51 AU) at 0.5 μL/min, 37.5 mM NaCit, and 2 mM AgNO_3_.

AgNP was analyzed by TEM (Fig. 4) using identical final reaction concentrations of 37.5 mM NaCit and 2 mM AgNO_3_ in batch (Fig. 4A) and on the microfluidic device (Fig. 4B). This showed that the AgNP tended to grow when exposed to the microfluidic environment as opposed to batch synthesis. Box and whisker plots show the average size in batch control of 31 nm (Fig. 4A) increases to 71 nm when synthesized on the microfluidic device at 0.5 μL/min. It seems important to mention that two size distributions can be seen in Fig. 4B labelled i. larger distribution (~ 71 nm) and ii. smaller distribution (~ 31 nm). The box and whisker plot in Fig. 4B is of the i. sized particles. In Fig. 4C, we can see the smaller particles coalescing around a larger particle. The box and whisker plot of Fig. 4C represents the smaller distribution of particles indicated in Fig. 4B ii. and the blue arrows in Fig. 4C.

The AgNP was also analyzed by SEM. The AgNP from the positive control experiment was viewed using the SEM to determine the size and morphology of the particles created as shown in Fig. 5A an optimized batch method for synthesis (37.5 mM NaCit and 1 mM AgNO_3_). The AgNP produced in the microfluidic device was also viewed using the SEM to determine if there was a difference in their size and morphology as compared to the positive control samples, as shown in Fig. 5B–D. A higher flowrate of 3 μL/min with 37.5 mM NaCit and 2 mM AgNO_3_ resulted in the smaller average particle size (~ 24 nm). However, increasing the NaCit concentration and reducing the flowrate to 1 μL/min resulted in a larger average AgNP (~ 400 nm). The AgNP imaged in Fig. 5C and D were created using the 75 mM NaCit concentration, resulting in much larger AgNP.

## Conclusion

This study presents evidence of the production of AgNP synthesized using a microfluidic chip using a minimal one-step method. The presence of AgNP was supported with the use of UV–Vis Spectroscopy, TEM, and SEM microscopy. We hope the production of AgNP in microfluidic chips will eventually lead to higher quality, accessibility, and more complex multi-materials. The use of predictive computer modeling can further be used to create a microfluidic device with higher efficiency mixing and incorporate the isolation of nanoparticles.

Our chip presents evidence that AgNP formation is confined to the central part of the channel, where the two reagent streams make contact due to the silver line formed. It remains unclear if the AgNP are sticking to one another, penetrating the PDMS, or are forming nucleation points for electroless chemical deposition. Our results suggest, but are not conclusive, that there is an interaction between the AgNP and the PDMS. We predict the AgNP are penetrating or forming within pores in the PDMS, which is known to be permeable. It could be that AgNP penetrates into the PDMS because of their very small size, and this creates the visible silver band. The trapped AgNP could have two fates: (1) continue to grow until they are released into the fluid stream or (2) AgNP grows in the PDMS pores to a large size and can no longer escape the PDMS pores, which could result in the formation of the silver line (Fig. 2). The reduction in flowrate results in size increases, which seems to indicate that AgNP is afforded the opportunity to grow larger by spending more time in the reductive environment of the microfluidic chip. The limitations of this study are due to the inability to analyze the microfluidic devices by SEM after AgNP synthesis. The chips are permanently stuck together by the oxygen plasma bonding process. Additionally, because the microfluidic chips must be run for a long period of time, the current experimental setup cannot determine if there is a time-dependent nature to the particle formation. It typically takes many hours to collect enough samples to analyze. Future directions will require a releasable microfluidic device, which can be removed and analyzed to determine what interaction is happening at the surface of the PDMS interface. We also will use additional chemicals to increase the nanoparticle yield. We can imagine several microfluidic functions that can be serially stacked for the chemical synthesis of complex multi-material nanoparticle composites not possible through other methods. A described device could be used to generate composite silver nanomaterial. In our device we would start with the nanoparticle seed formation using the described microfluidic mixer, followed by diffusion separation followed with mixing with cationic iron or gold [25–27]. In this way, we can create more complex materials for anti-viral/bacterial scaffolding and pharmaceutical development.
